# Mountain Toothache

**Published:** 1900-04-15

**Authors:** 


MOUNTAIN TOOTHACHE.—M. Hofner, of Zurich, Switzerland, says that the workmen on the railroad in process of construction up the Jungfrau, at the end of eight or ten days after commencing work, are attacked with severe toothache, accompanied by swelling of the gums and inflammation in the cheeks. The teeth are exceedingly sensitive to pressure, and mastication is accomplished with great difficulty. The pain increased up to the third day and gradually decreased until it entirely ceases at the end of six or seven days, with no troublesome sequelæ of any kind. The trouble is not recurrent. It is attributed to the high altitude, 3,000 meters.

---

Dr. A. W. McCandless, in *Dental Review*, March, page 228, very strongly recommends 25 percent platinum solder for all porcelain crown and bridge-work, to secure strength and stability. He also advocates setting crowns and bridges with gutta-percha, to give elasticity and to prevent fracture from sharp occlusions. It also makes it easier to remove for repair when necessary.

